# Editorial: T-cell directed therapies in multiple myeloma

**DOI:** 10.3389/fonc.2023.1206475

**Published:** 2023-06-16

**Authors:** Joshua Richter, Larry D. Anderson, Karthik Ramasamy, Adriana Rossi

**Affiliations:** ^1^ Tisch Cancer Institute, Icahn School of Medicine at Mount Sinai, New York, NY, United States; ^2^ Myeloma, Waldenstrom’s, and Amyloidosis Program, Simmons Comprehensive Cancer Center, University of Texas (UT) Southwestern Medical Center, Dallas, TX, United States; ^3^ Radcliffe Department of Medicine, Oxford University Hospitals National Health Services (NHS) Foundation Trust, Oxford, United Kingdom; ^4^ Oxford Translational Myeloma Centre, Nuffield Department of Orthopaedics, Rheumatology and Musculoskeletal Sciences (NDORMS), University of Oxford, Oxford, United Kingdom

**Keywords:** myeloma, bispecific Ab, CAR-T, immunotherapy, T-cell redirection

In the beginning, our approach to treating patients with multiple myeloma (MM) was just like any other malignancy: systemic cytotoxic chemotherapy. The key players in this first epoch were alkylators such as melphalan and cyclophosphamide and combinations such as VAD (vincristine, Adriamycin, and dexamethasone). In the early 2000s, we began the transition to the 2^nd^ epoch: the “novel therapies”. Initially dominated by immunomodulatory drugs such as thalidomide and lenalidomide as well as proteasome inhibitors such as bortezomib and carfilzomib and ultimately culminating, circa 2015, in monoclonal antibodies targeting anti-CD38 Daratumumab, Isatuximab) and BCMA (Belantamab mafodotin). In addition, drugs with new mechanisms of action, Selinexor and Melflufen were made available. On March 26, 2021, we had our first approval (idecabtagene vicleucel) (1) which thrust us into the 3^rd^ epoch: t-cell redirection therapy (TCRT).

At the time of writing this editorial, there have been 3 FDA approved TCRTs: the two B-cell maturation antigen (BCMA)-targeted chimeric antigen receptor T-cell (CAR-T) products, idecabtagene vicleucel and ciltacabtagene autoleucel (2), and the BCMA targeted bispecific antibody (bisab) teclistamab (3). Furthermore, there are currently 2 more bisabs that have been filed to the FDA and are awaiting potential approval: an additional BCMA-targeted bisab, elranatamab (4), and the G protein-coupled receptor class C group 5 member D (GPRC5d) -targeted talquetamab (5).

The 2^nd^ epoch showed a number of approvals of drugs (either alone or in combination with dexamethasone) in patients that were refractory to all previously available therapies and typically yielded overall response rates of 20%-30%. Steroids were systematically added to this program with the augmentation of response and adverse events. This 3^rd^ epoch, however, to date has led to approvals of therapies with response rates ranging from 63% to over 90%. This can be seen in **Table 1**.

**Table 1:** Approvals of myeloma therapies in the late relapse.

EPOCH	Drug	median prior lines of therapy	ORR (%)	Trial
2	belantamab	7	31	DREAMM-2 (6)
2	carfilzomib	5	23.7	PX-171-003-A1 (7)
2	daratumumab	5	29.2	SIRIUS (8)
2	melflufen	5	29	HORIZON (9)
2	pomalidomide	5	31	MM-003 (10)
2	selinexor	7	26	STORM (11)
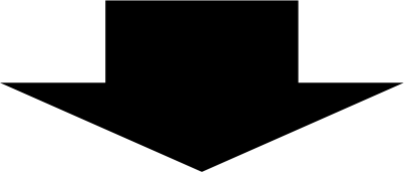				
3	cilta-cel	6	94	Cartitude-1 (12)
3	ide-cel	6	73	Karmma-1 (13)
3	teclistamab	5	63	Majestic-1 (14)

Furthermore, although the number of median prior lines of therapy is similar between these trial groups, the nature of those prior lines and drug exposures is extremely different. As increasingly effective regimens such as Dara-RVD have been rising in level of utilization in front-line therapy for myeloma, patients can be triple-class exposed entering their 2^nd^ line of therapy, and triple-class refractory not long after that. This makes the high rates of response of the TCRTs even more impressive.

This Research Topic outlines the major issues surrounding TCRTs: current landscape, future directions, and management of TCRT-specific adverse events. Boussi et al. provide a great framework for immunotherapy and its broad impact on MM. They set the stage for myeloma as an archetype for immune dysregulation. As an immune-based malignancy, to some degree, all therapeutic approaches can be classified as “immunotherapy”. This includes but is not limited to approaches such as autologous and allogeneic stem cell transplantation, cellular therapies utilizing T-cells and NK-cells, and checkpoint inhibition and vaccine-based strategies. Cho et al. and Abebe et al. go on to describe the current standards in TCRTs: bisabs and CAR-Ts. The clinical trial landscape is flush with bisabs of all varieties, the majority of which target BCMA, with promising novel targets including FcRH5, GPRC5d, and CD38. CARTs such as cilta-cel and ide-cel have shown deep and rapid responses in heavily refractory patients with emerging data from Karmma-3 (15) and Cartitude-4 (16) showing impressive responses in earlier lines, including double-class refractory patients.

Banerjee et al. and Alabanza et al. give us a glimpse as to where the future is headed for TCRTs. Current approaches towards the optimization of CARTs include a variety of tactics such as modification of the tumor cell by increasing BCMA expression. Other options focus on modification of the CAR such as dual-targeting platforms or “armored” CARs. Further still, some approaches are looking to alter the immune milieu through a variety of manipulations in efforts to provide the optimal setting to allow the exogenous CAR T-cells to kill the endogenous plasma cells on a backdrop of a supportive immune environment. Lastly, allogeneic products using healthy donor T cells are also under investigation.

As TCRTs “redirect” T-cells away from their planned function and towards malignant plasma cells, the major resultant impact is increased in infectious complications. John et al. explores some of the current knowledge surrounding therapy-acquired secondary immunodeficiency (SID). There are significant efforts underway to better understand the depth and nature of this SID as well as the optimal strategies to reduce its impact such as intravenous immune globulin support, prophylactic anti-infectives, and limited duration exposure to TCRTs.

The new paradigm of TCRTs provides unprecedented levels of response. At the same time, our optimization of their impact on efficacy and toxicity has yet to be fully defined. That said, these new therapies may be part of a future prospect of either a) fixed-duration therapy in a world where the current paradigm is “treat until progression” or b) potentially curative approaches!

## Author contributions

All authors listed have made a substantial, direct, and intellectual contribution to the work and approved it for publication.
